# Coronavirus disease 2019: Repeated immersion of chlorine-containing disinfectants has adverse effects on goggles

**DOI:** 10.3389/fpubh.2023.1016938

**Published:** 2023-01-20

**Authors:** Xiao-bo Zhang, Yu-ling Wei, Gang Zhao, Mei He, Jun Sun, Wen Zeng

**Affiliations:** ^1^Department of Spine Surgery, Honghui Hospital, Xi'an Jiaotong University, Xi'an, Shaanxi, China; ^2^Department of Respiratory, Honghui Hospital, Xi'an Jiaotong University, Xi'an, Shaanxi, China; ^3^Department of Operating Anesthesia Room 1, Honghui Hospital, Xi'an Jiaotong University, Xi'an, Shaanxi, China; ^4^Department of Pediatric Orthopaedic Hospital, Honghui Hospital, Xi'an Jiaotong University, Xi'an, Shaanxi, China; ^5^Department of Quality Control, Honghui Hospital, Xi'an Jiaotong University, Xi'an, Shaanxi, China

**Keywords:** coronavirus disease 2019, chlorine-containing disinfectants, repeated immersion, goggles, adverse effect

## Abstract

**Introduction:**

During COVID-19, some front-line personnel experienced varying degrees of eye discomfort due to the use of goggles repeatedly disinfected with chlorine-containing disinfectant.

**Methods:**

The eye damage information of 276 front-line personnel who used goggles in a hospital from October 1, 2021, to December 1, 2021, was collected by filling out a questionnaire. To study the effect of chlorinated disinfectants on goggles, we immersed the goggles in the same volume of water and chlorinated disinfectant buckets. We tested the light transmittance, color and texture, and airtightness of the goggles at different times (1, 3, 12, 24, 36, 48, 60, 72, 96, 120, 144, 168, 192, 216, 240, and 268 h). In addition, we detected where chlorinated disinfectant remained in the goggles by using disinfectant concentration test paper.

**Results:**

60 (21.82%) people experienced dry eyes, stinging pain, photophobia and tearing, conjunctival congestion, eyelid redness, and swelling. After treatment or rest, the patient's ocular symptoms were significantly relieved within 3 days. With the extension of disinfection time, the light transmission of the lenses gradually decreased, and the light transmission reduced when immersion occurred at 216 h. After 72 h of disinfection, the color of the goggle frame began to change to light yellow, the texture gradually became hard and brittle, and the color became significantly darker at 268 h of disinfection. The airtightness of the goggles began to decrease after 168 h of disinfection, the airtightness decreased substantially at 268 h, and the shape changed significantly. In addition, the concentration test paper results show that the disinfection solution mainly resides in the goggle frame seam and goggles' elastic bands' bundle.

**Conclusions:**

Repeated chlorine disinfectant disinfection will reduce the effectiveness of goggles protection and damage front-line personnel's eye health.

## 1. Introduction

The Coronavirus disease 2019 (COVID-19) has caused tremendous economic losses worldwide, including handwashing, mask-wearing, and physical distancing as effective measures to prevent the spread of the epidemic (1). The COVID-19 pathogen is severe acute respiratory syndrome coronavirus 2 (SARS-CoV-2), which can be transmitted through the conjunctiva (2). The virus may be transmitted from the eye surface to the new host through contact with eye mucous membrane, tears or subsequent pollutants. Eye protection equipment such as goggles or masks shall be used as part of standard personal protective equipment (3). Therefore, front-line personnel must wear goggles to avoid infection by infectious splashes. Obviously, personal protective equipment covering more body parts and standard wear and tear prevention techniques can provide better protection (4). However, for various reasons, many countries and institutions are faced with the challenge between appropriate protection and rationing to reduce supply (5). Disposable goggles can be reused when necessary personal protective equipment (PPE) is lacking (6). When the necessary personal protective equipment (PPE) is lacking, disposable goggles will be disinfected and reused.

There are various ways to disinfect goggles, including disinfectant spray or immersion, preprogrammed automatic spray cleaning and disinfection machines and UV disinfection, but there is no unified opinion (7). In China, chlorine-containing disinfectants are widely used to disinfect various epidemic environments and PPE because of their excellent disinfection effect, easy access, and low price, which can effectively block the epidemic's spread. However, there is no unified specification for the goggle use duration. During our work, we found that some front-line personnel experienced varying degrees of eye discomfort during COVID-19, which we attributed to using goggles repeatedly disinfected with chlorine-containing disinfectants. After reviewing the literature, there are no objective criteria or reports for goggle replacement. Therefore, we aimed to analyze the adverse effects of goggles disinfected with chlorine-containing disinfectants on healthcare workers to provide a reference for front-line personnel to fight COVID-19 more efficiently and safely.

## 2. Methods

### 2.1. Inclusion and exclusion criteria

All front-line personnel adheres to personal protective equipment standards (8). To study eye damage, we counted the eye damage of front-line personnel who had used goggles in a hospital in Xi'an by completing a questionnaire. The inclusion criteria were all front-line personnel who had used goggles between October 1, 2021, and December 1, 2021. The exclusion criteria were those with recent or previous eye problems and incomplete information in the questionnaire. Our study methodology is repeatable.

### 2.2. Disinfection process of goggles

We used medical goggles (Shenzhen careful eye technology Co., Ltd.) with chlorine disinfectant (effervescent disinfectant tablets type II, Shandong Lierkang Medical Technology Co., Ltd.), whose main component is trichloroisocyanuric acid. First, the goggles were disinfected with 1,000 mg/L disinfectant for 30 min, fished out and placed in clear water for 30 min, rinsed under running water for 5 min, carefully wiped clean with 75% medical alcohol, and used after drying. In contrast, the clear water group was immersed in a bucket with an equal volume of water. The cleaning staff changed the chlorinated disinfectant and the fresh water every 24 h.

### 2.3. Light transmission

After wearing the goggles, we tested the goggles' light transmission by observing the water's transparency in the container (Supplementary Figure 1).

### 2.4. The color and texture

The color and texture changes in the plastic on both sides of the goggles were observed at different time points (1, 3, 12, 24, 36, 48, 60, 72, 96, 120, 144, 168, 192, 216, 240, and 268 h) after immersion in chlorine disinfectant. Three people were observed to reduce the variation due to different individuals.

### 2.5. The airtightness

We tested the goggle airtightness in the following three ways. The first method was to observe the face fit from the side after the experimenter wore the goggles. Another person observed the goggles from the side. The second method was to place cotton wool inside the goggles, squeeze the simple airbag along the edge of the goggles, and observe whether the cotton wool moved inside the goggles. The third method was to test whether the suitability of the elastic bands of the goggles decreased.

### 2.6. Detect residual disinfectant

The concentration test paper (L-1 disinfectant concentration test paper, Shandong Lierkang Medical Technology Co., Ltd) was used to detect the goggle lenses, frame lens joints, frames, goggles and elastic bands bundles and elastic bands. The color of the test paper was observed through the indicator card of the disinfectant concentration test paper (Supplementary material 2) to judge whether there was residual disinfectant in the goggles and the residual position.

### 2.7. Statistical analysis

The data were analyzed using SPSS (version 26.0). The results were expressed as the mean ± standard deviation. As the data in this paper collected the eye symptoms of front-line personnel using goggles in questionnaires, no statistical analysis of differences was conducted.

## 3. Results

### 3.1. General information

**Figure 1:**
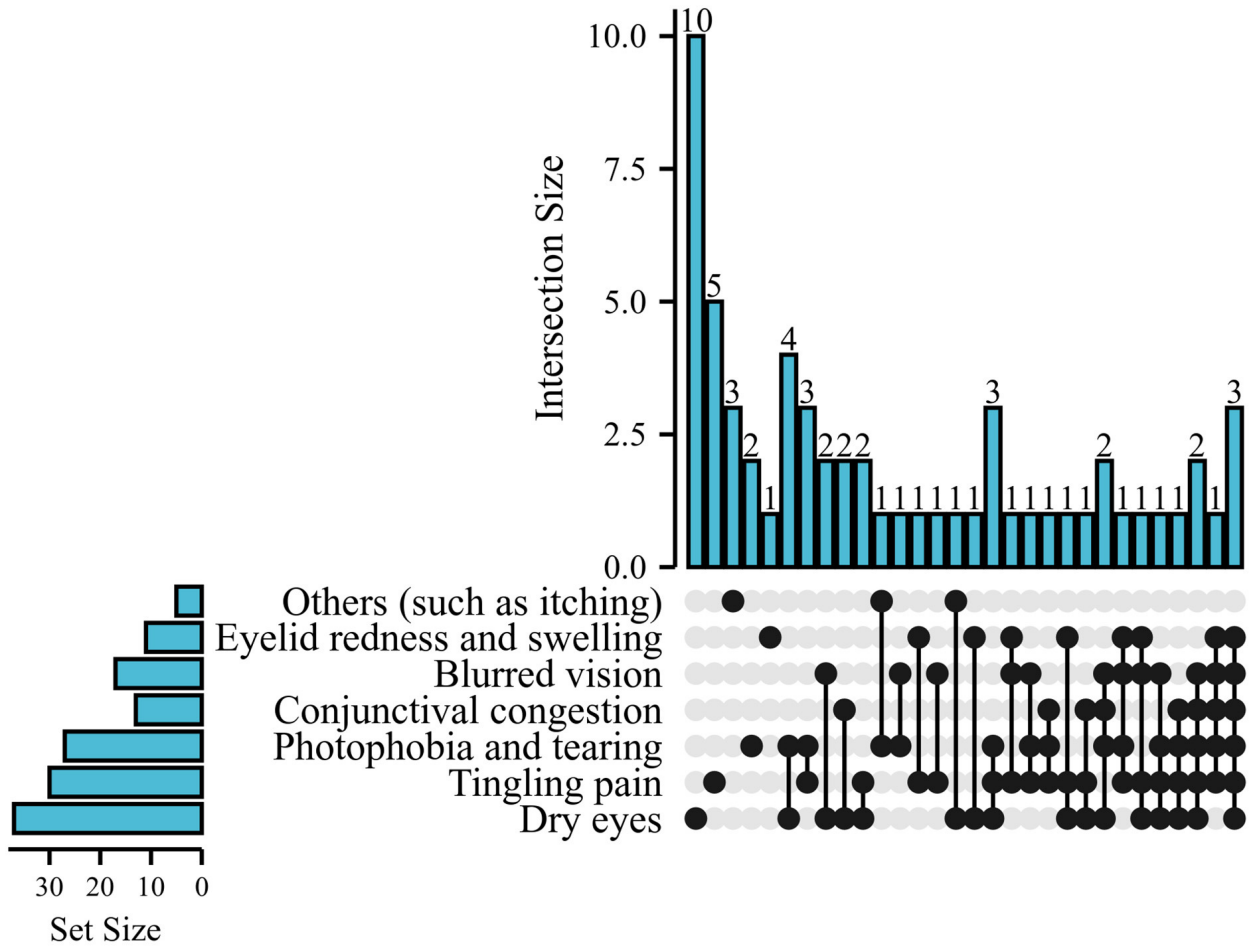
Summary of the proportion of eye symptoms, including dry eyes, stinging pain, photophobia and tearing, conjunctival congestion, eyelid redness, and swelling, with dry eyes (61.67%) and stinging pain (50.0%) being the main symptoms.

A total of 298 people completed the questionnaire, 17 were excluded due to previous eye disease, and 6 were excluded due to incomplete questionnaire completion. Ultimately, 275 people were included in this study. Among them, 51 (18.55%) people were males, 224 (81.45%) people were females, 108 (39.27%) people were doctors, 166 (60.36%) people were nurses, and 1 (0.36%) person was cleaner, shown in Table 1. During the work period, all healthcare workers were not infected with COVID-19, and 60 (21.82%) had ocular symptoms, including 37 dry eyes, 30 stinging pain, 27 photophobia and tearing, 17 blurred vision, 13 conjunctival congestion, and 11 eyelid redness and swelling, with dry eyes and stinging pain as the main symptoms. Only 21 (35.0%) had single-eye symptoms among these people with eye damage, as shown in Figure 1. After the occurrence of eye damage, 47 (78.33%) people were significantly relieved after observation and rest, 1 (1.67%) person was rinsed with normal saline, and the remaining 12 (20.00%) people were treated with drugs (such as levofloxacin). Their eye discomfort symptoms were significantly relieved within 3 days.

**Table 1:** General information.

	**Male (51)**	**Female (224)**
Age	32.04 ± 7.47	31.15 ± 6.50
**Occupation**		
Doctor	36	72
Nurse	15	151
Cleaner	0	1
Eye disease	8	52
**Therapeutic method**		
Observation	8	39
Normal saline flushing	0	1
Drug treatment	0	12

### 3.2. Reduce light transmission

**Figure 2:**
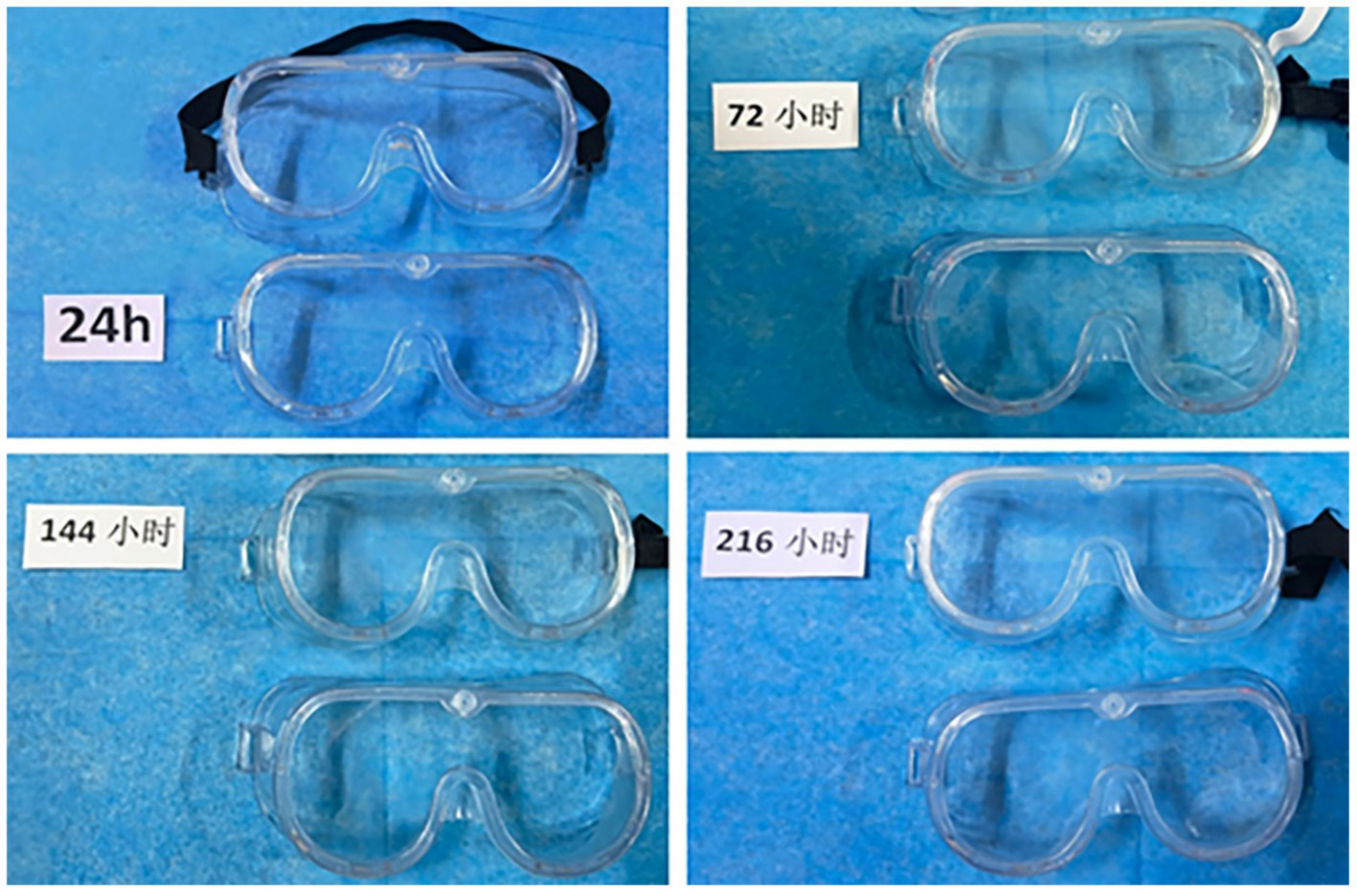
The light transmittance change of goggles immersed in disinfection at different times. Goggles with black bands are the disinfectant group, and goggles without bands are the clear water group.

After continuous disinfection of the goggles by immersion, the light transmission of the clear water and disinfectant groups decreased to different degrees. After immersion for 216 h, the lenses of the clear water group were still translucent, but the lenses of the disinfectant group became yellow and darker, as shown in Figure 2.

### 3.3. The color and texture changed

**Figure 3:**
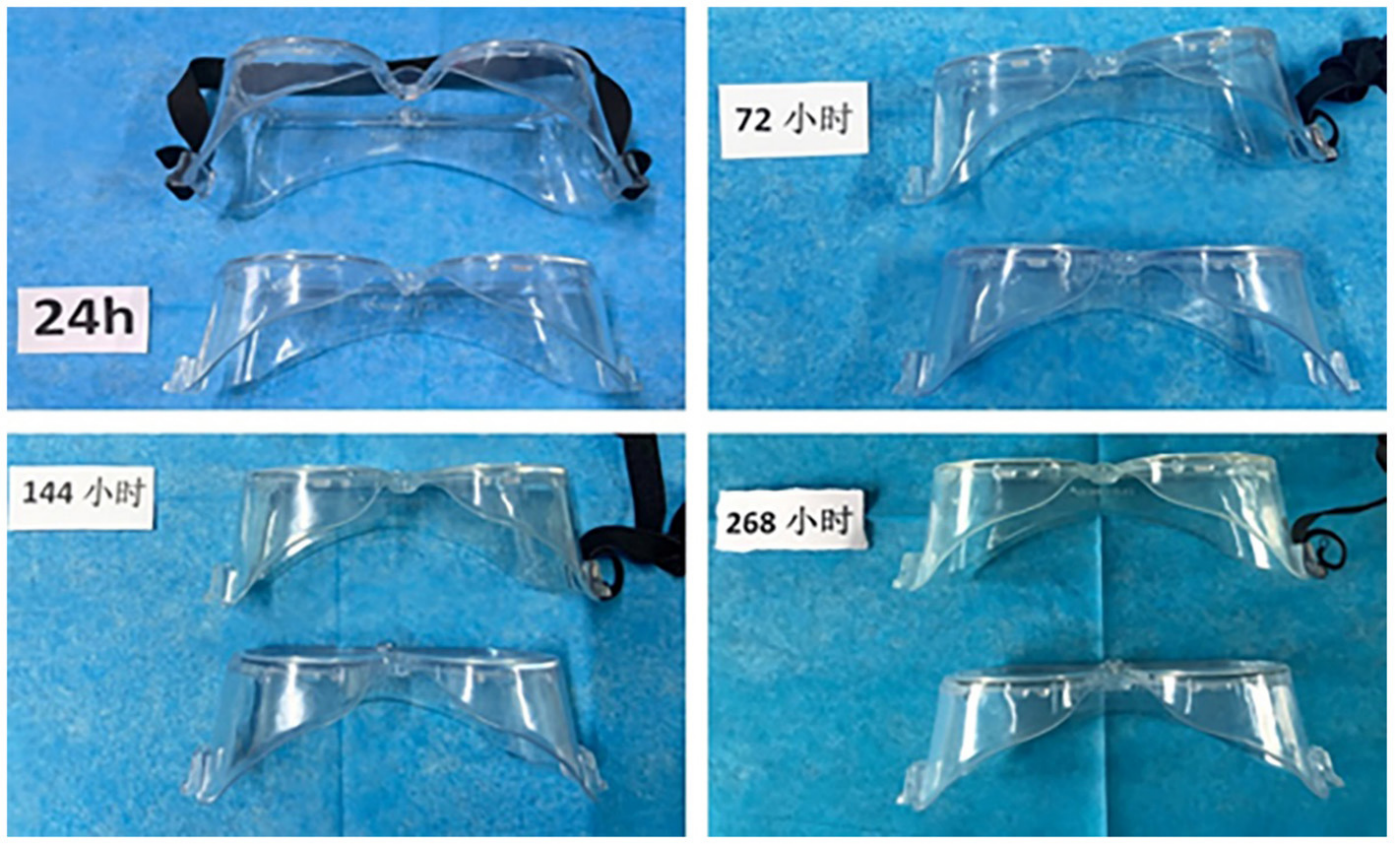
After immersion in chlorine-containing disinfectants, the goggles' frame gradually changed to light yellow with time. Goggles with black bands are the disinfectant group, and goggles without bands are the clear water group.

With prolonged disinfection time, the color of both sides of the goggles gradually changed. After 72 h of disinfection, the color of the goggles gradually changed to light yellow, and the texture gradually became hard and brittle. After immersion for 268 h, the black elastic bands of the disinfectant gradually changed to light yellow with time (Figure 3). In addition, compared with the water, the goggles in the disinfectant are more rigid and brittle.

### 3.4. The airtightness reduced

**Figure 4:**
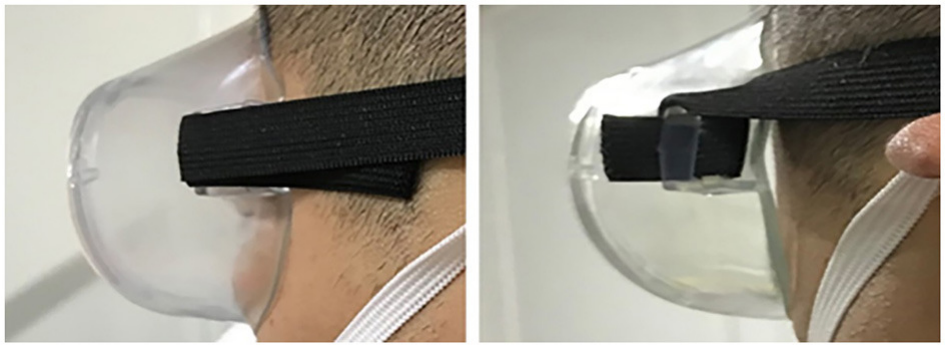
The goggles' shape changes with the time of disinfection by disinfectant immersion.

The air tightness of goggles in the clear water group was greater than that in the disinfectant group, suggesting that liquid immersion may have some adverse effects on the goggles. According to our observation, the goggle airtightness started to change after immersion for 168 h. After immersion for 268 h, the goggles are partially deformed and do not fit the face completely (Figure 4). Second, the cotton wool moved when the goggle edge squeezed the simple airbag (Supplementary material 3). Finally, the elastic bands appeared slack. In conclusion, when the goggles were immersed with disinfectant for a long time, the airtightness was significantly reduced, thus losing the protective effect on the eyes.

### 3.5. Disinfectant residue in goggles

**Figure 5:**
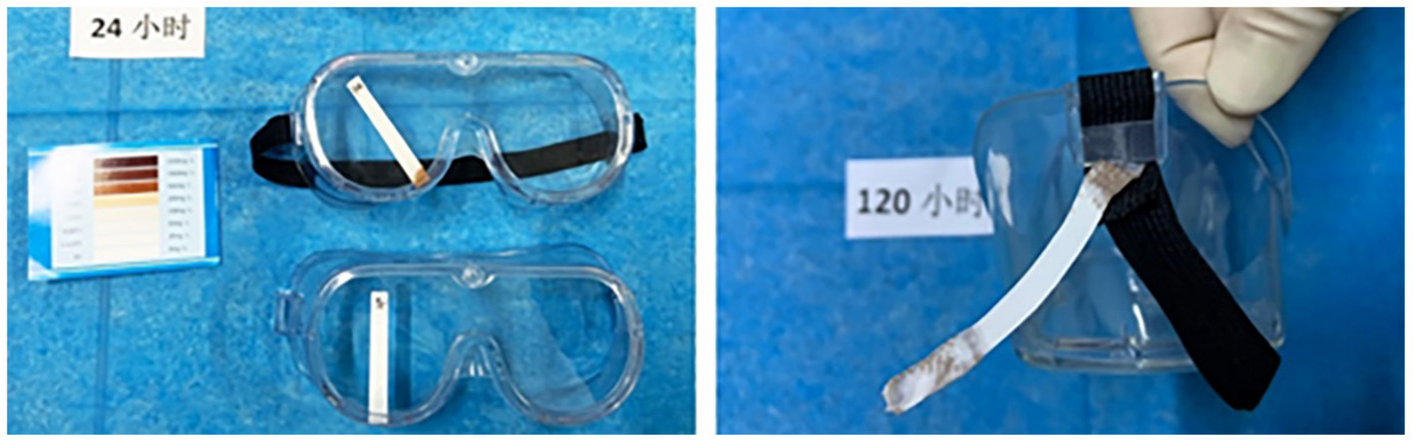
The disinfection solution mainly resides in the goggle frame lens seam and goggles' elastic bands' bundle.

Changes in the color of the concentration test paper were found in the goggle frame seam and goggles' elastic bands' bundle (Figure 5), suggesting that a small amount of disinfectant remained in these positions.

## 4. Discussion

Studies have shown that ocular symptoms, including dry eye, are relatively common in COVID-19 patients and can occur before respiratory symptoms, but the extent of ocular involvement is largely ignored by clinicians (9, 10). Eye tissue and eye fluid may be the potential source and route of COVID-19 infection. Some anatomical and physiological characteristics of the eye surface may promote its role as a gateway to respiratory tract infection and a potential site for virus replication (11). Eye protection with goggles is problematic due to “fogging”. It is recommended to use filter type eye mask (12) or non fogging goggles (13) for eye protuction, disinfect strictly when resuse, to prevent eye transmission.

Trichloroisocyanuric acid is an oxidizing and chlorinating agent with a strong disinfection effect. It has highly effective chlorine content, stable storage and transportation, convenient molding and use, high sterilization and bleaching power, long adequate chlorine release time, safety, and nontoxicity. Therefore, its development and research have received attention from various countries. Recently, Jiang et al. made a novel nan-efficient disinfectant by loading trichloroisocyanuric acid into graphene oxide with the blending method (14). As front-line personnel at COVID-19, goggles disinfected with chlorine-containing disinfectants are often used due to necessary PPE scare. Nevertheless, the goggle replacement standards are not uniform. It depends mainly on the subjective experience of front-line personnel. Therefore, we investigated the effect of trichloroisocyanuric acid on goggles by these indicators.

The goggles' light transmission is a vital reference factor for the wearer on whether to replace the goggles. We found that disinfectants have a more significant effect on the light transmission of goggles than water, with the lenses becoming yellow and darker. At the same time, the continuous disinfectant immersion caused the goggle frames to change color and the goggle frames to become more rigid and brittle in texture, which affected the work efficiency. In terms of airtightness, the results suggest that liquid immersion causes the goggles to be less airtight, but disinfectant immersion significantly reduces the airtightness of the goggles. The change in the shape of the goggles led to goggles not fitting the face entirely, increasing the risk of eye infection with SARS-CoV-2. In addition, with prolonged disinfection time, the elasticity of goggles' elastic bands gradually decreased, indirectly leading to goggle airtightness decrease. While the elastic bands are too tight, they not only enhance the protection effect but also damage the skin, produce fog, and lead to local skin indentation. In conclusion, the disinfected goggles became yellowish and darker, the airtightness decreased, and some locations had disinfectant residues.

Our study showed that approximately one-fifth of the personnel showed symptoms of eye damage, which manifested as dry eyes, stinging pain, photophobia and tearing, and conjunctival congestion. Therefore, wash the goggles with disinfectant after disinfecting them thoroughly to remove harmful residues. However, these disinfectants remained in the goggle frame and lens joints and the goggles and elastic bundles despite water immersion, running water flushing, and 75% medical alcohol wiping after disinfection. When manual cleaning is used, adequate and extended rinsing time under running water is required. Still, even prolonged rinsing time also be challenging for goggle-specific locations such as the lens seam of the frame. In addition, the goggles are manufactured from polycarbonate polymer material, while trichloroisocyanuric acid is a potent oxidizing and chlorinating agent. Repeated immersion disinfection of goggles may be subject to corrosion and oxidation, resulting in changes within the material. When wearing eyes in a closed space, goggles' internal residual disinfectant in specific humidity and temperature conditions are volatile, thus bringing harm to the eyes.

In response, we propose several possible improvements as follows. First, we suggest improving the disinfection process. The standard programmed operation can reduce the labor intensity of cleaning personnel, shorten the time of staff exposure to contaminated instruments, and reduce occupational hazards. Recently, Wang et al. concluded that machinery sterilization set at 70°C for 30 min has better cleaning and sterilizing effects for reusable medical goggles (7). However, whether this disinfection method is effective and widespread remains to be further confirmed. In other words, in times of scarce resources, institutions may turn to alternative methods of preserving and reusing goggles. In addition, UV radiation, a well-known technique for inactivating microorganisms and viruses, offers several advantages over thermal sterilization or chemical disinfectants. The whole process can be performed automatically for disinfecting liquids, surfaces, air and rooms, and it is energy efficient. Previous studies have shown that UV radiation is effective against coronaviruses, and the inactivation of SARS-CoV-2 by UV irradiation may be a reliable method (15, 16). Please note that the goggles must be replaced on time after effective disinfection and repeated multiple uses.

Second, the goggles' shapes can be improved by using additive manufacturing technologies to reduce the slit and the possibility of disinfectant residue. In the media, on different video-sharing platforms and social media sites, many publications, articles, and videos have shared how to produce masks, face shields, or goggles intended for civilian or professional healthcare-related use to improve the supply chain system (17). However, critical evaluation is essential to provide detailed insight into these products and minimize potential risks and hazards to avoid the preventable loss of healthcare workers or patients due to the improper design or use of these devices (18). Celik et al. designed a medical face shield that is competitively lighter, relatively more ergonomic, and easy to use (19). Additive manufacturing technology can assemble them without extra seams (such as elastic bands, softening materials, and clips). Meanwhile, 3D-printed PPE is reported to be reusable, which offers a possible way to develop goggles (20). Finally, to protect the personnel's eye health, we recommend using disposable goggles as much as possible when conditions permit.

## 5. Conclusions

Wearing goggles disinfected with chlorinated disinfectants reduces goggle protection effectiveness and increases eye damage risk, which can be improved by improving disinfection methods, changing the shape of the goggles, or simply using disposable goggles. The study had limitations, such as a small sample size and a single-center study.

## Data availability statement

The raw data supporting the conclusions of this article will be made available by the authors, without undue reservation.

## Ethics statement

Ethical review and approval was not required for the study on human participants in accordance with the local legislation and institutional requirements. Written informed consent for participation was not required for this study in accordance with the national legislation and the institutional requirements. Written informed consent was obtained from the individual(s) for the publication of any potentially identifiable images or data included in this article.

## Author contributions

X-bZ drafted the manuscript. JS supervised the data collection. WZ conceived the study design and contributed to the revision. All authors contributed to the writing of the manuscript.

## Supplementary material

Supplementary Figure 1 The transparency of the water in the container after wearing the goggles.

Supplementary Material 2 Observe the color of the test paper through the indicator card of the disinfectant concentration test paper to judge whether there is residual disinfectant in the goggles and the residual position.

Supplementary Material 3 The cotton wool moved when the goggle edge squeezed the simple airbag.
