# Supplementary figures and images for: Coronavirus disease 2019: Repeated immersion of chlorine-containing disinfectants has adverse effects on goggles
Source: Front Public Health. 2023 Jan 20;11:1016938. doi: 10.3389/fpubh.2023.1016938 (PMC9895403; doi:10.3389/fpubh.2023.1016938)

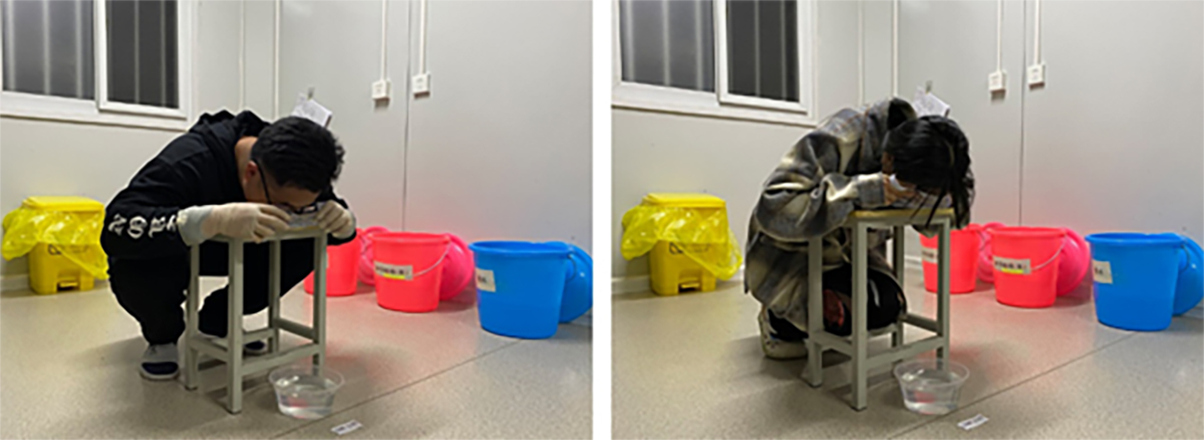

Supplement: Supplementary Figure 1 — The transparency of the water in the container after wearing the goggles. [file Image_1.tif]

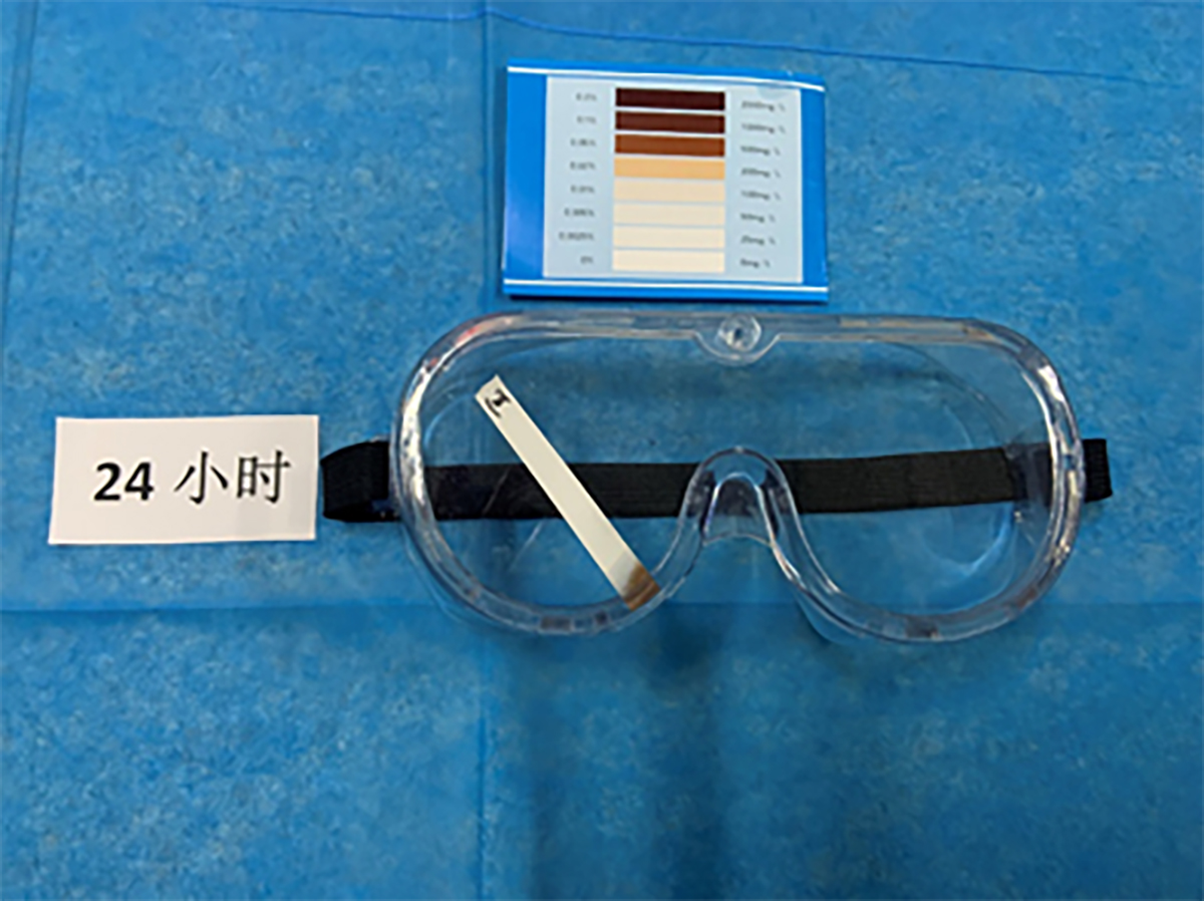

Supplement: Supplementary Material 2 — Observe the color of the test paper through the indicator card of the disinfectant concentration test paper to judge whether there is residual disinfectant in the goggles and the residual position. [file Image_2.tif]
